# Trajectories of decline on instrumental activities of daily living prior to dementia in persons with mild cognitive impairment

**DOI:** 10.1002/gps.5426

**Published:** 2020-09-17

**Authors:** Simon Cloutier, Howard Chertkow, Marie‐Jeanne Kergoat, Isabelle Gélinas, Serge Gauthier, Sylvie Belleville

**Affiliations:** ^1^ Institut universitaire de gériatrie de Montréal Montreal Quebec Canada; ^2^ Lady Davis Institute Montreal Quebec Canada; ^3^ Davis House School of Physical and Occupational Therapy McGill University Montreal Quebec Canada; ^4^ Alzheimer Disease Research Unit McGill Center for Studies in Aging Montreal Quebec Canada

**Keywords:** activities of daily living, Alzheimer's disease, mild cognitive impairment, trajectories

## Abstract

**Objectives:**

The main objective was to determine the trajectory of instrumental activities of daily living (iADL) decline in persons with mild cognitive impairment (MCI) who progressed towards dementia relative to persons with MCI who remained stable.

**Methods/Design:**

At study entry, 121 participants met criteria for MCI. Based on the follow‐up, 47 participants later converted to dementia and were identified as progressors. Sixteen participants, identified as decliners, presented a significant cognitive decline but did not reach the criteria for dementia within the study timeframe. Stable MCI remained cognitively stable during the 5‐year follow‐up; *n* = 58. Participants completed a yearly assessment using clinical tests/questionnaires, neuropsychological measures, and functional autonomy assessment until they met criteria for dementia. The average number of months for the follow‐up was 34.

**Results:**

Many years of stable performance followed by an accelerated decline just prior to diagnosis, was observed for complex activities for progressors. No change was found for stable MCI and a gradual linear decline characterized decliners. The *housekeeping‐related activities* component showed a linear decline in progressors and did not change in stable and decliner MCI. We found a predictive model that includes significant predictors of dementia conversion with a high diagnostic accuracy the following year (area under the curve = 0.94 [95% confidence level; lower bound: 0.87, upper bound: 1]).

**Conclusions:**

It is critical to assess iADL that reflect complex activities in the evaluation of MCI individuals as their impairment, combined with change on cognitive markers, indicates a higher risk of dementia progression 1 or 2 years later.

**Abbreviations**
GDS: geriatric depression scale
MATTIS: mattis dementia rating scale
MMSE: mini mental state examination
PCA: principal component analysis
WAIS‐R: wechsler adult intelligence scale‐revised

## 1 INTRODUCTION

Dementias are a group of major neurocognitive disorders that are defined by a decline from previous levels of functioning and a cognitive impairment involving at least two cognitive domains.^1^ Alzheimer's disease (AD) is the most common type of dementia. AD is characterized by an insidious onset and is known to have a long prodromal phase during which cognitive symptoms are mild or absent. The term mild cognitive impairment (MCI) is used to describe individuals whose performance on neuropsychological tests is abnormal for their age and education level. Persons with MCI don't meet the criteria for dementia because the cognitive deficits are not severe enough to significantly interfere with activities of daily living (ADL).^2^, ^3^, ^4^ Yet, the disease is progressive and as cognitive deficits accumulate, patients may experience growing problems in their ability to perform activities of daily living (ADL), particularly instrumental ADL (iADL) such as financial management, the use of telephone, or cooking, as these require more advanced skills.^5^

**Key Points**

- Mild cognitive impairment (MCI) progressors experience many years of stable performance of instrumental activities of daily living (iADL) but they show a rapid decline of complex iADL about two years prior to the clinical diagnosis of dementia, a pattern which is not found in stable MCI
- Performance on iADL declines in late MCI, especially for complex tasks, and observing a change on these activities, particularly when combined with lower performance on neuropsychological tests, signals an imminent progression to dementia in the following one or two years

There is evidence that MCI participants are significantly more impaired on iADL than healthy older adults^6^, ^7^, ^8^; for a review see Ref.^9^ Difficulties with executive functions, which include the cognitive abilities used to control actions and goal‐oriented behavior, have been consistently associated with difficulties in performing iADL in early dementia.^10^, ^11^, ^12^, ^13^ Similar difficulties can occur for MCI individuals, since executive functions are already impaired during that phase.^14^, ^15^, ^16^ It has been found that individuals with MCI, in comparison to controls, are impaired on complex iADL related to frontal/executive functioning (e.g., keeping appointments and managing belongings).^17^ Even when performance scores on iADL scales are similar to controls, some subtle but still notable difficulties were found during MCI, such as a reduced speed in telephone use, or medication management.^18^ Subtle change in the ability to perform iADL have been observed up to 10 years before the clinical diagnosis of dementia.^19^

Given that iADL are impaired early in the disease process and may index future decline, it is critical to know the moment at which those difficulties appear. It is also critical to describe their trajectory and how they change over time because change in functions is often considered a more sensitive and specific marker of future decline than performance level at a single timepoint. Since cognitive deficits increase in severity and breadth during the MCI phase,^20^, ^21^ the magnitude of the functional impact is likely to change as well.

In AD, the cognitive trajectories are characterized by a rapid and severe decline of episodic and working memory just prior to diagnosis.^20^, ^22^ Since the decline in iADL is due to emerging cognitive deficits^23^ in AD and is especially associated with executive functions and memory capacities, their impairment may follow a similar trajectory, that is years of stable performance followed by a rapid decline just prior to the diagnosis.

There are few longitudinal studies^24^, ^25^, ^26^ investigating the trajectory of iADL change in the years preceding the AD diagnosis and as a result, little is known regarding the way iADL impairment unfolds over time. Furthermore, no study, to our knowledge, has examined the natural history of the decline in iADL for a clinical cohort of MCI individuals as a function of whether they progressed to dementia or remained stable. As not all MCI will progress to dementia, it is important to compare the trajectory in those who progressed to a dementia diagnosis (MCI progressors) relative those who did not progress (MCI nonprogressors).

Thus, the objective of this study was to assess the trajectory of decline in iADL for MCI progressors and compare this trajectory with the one found in MCI nonprogressors. This was done using a mixed model analysis with polynomial regressions to assess with more precision the way the ability to perform iADL changes over time. We hypothesized that a significant decline on iADL would be found for the progressors, whereas the nonprogressors would remain stable on their ability to perform these activities. A second objective was to determine if combining information on iADL and cognitive performance may offer a sensitive model to predict future progression.^27^ Since impairment on iADL appears to be associated with executive and memory deficits, we hypothesized that the combination of the performance in these cognitive domains with the iADL score would be significant predictors of AD progression.

## 2 MATERIALS AND METHODS

### 2.1 Design

Patients were recruited consecutively from memory clinics and were identified as meeting criteria for MCI by experienced clinicians (HC, MJK, SG). They were then referred to participate to a longitudinal study on cognition in MCI, which lasted 8 years.^28^ At study entry and at yearly follow‐up, participants completed a comprehensive clinical and neuropsychological examination. All measures were taken in a single testing session. The referring clinicians determined the clinical status on follow‐up assessments, independent from the experimental tests, and experimental follow‐up was interrupted the year a patient received a diagnosis of dementia. Thus, the last assessment corresponds to the year of dementia diagnosis.

T0 represent the year of conversion, that is, the year when participants received the diagnosis of dementia for those who declined to AD, T‐1, T‐2, T‐3, and T‐4 correspond to respectively the data collected 1, 2, 3, and 4 years prior to diagnosis. The last year of evaluation is labeled as T0 for nonprogressors (decliners and stable MCI) and in this case, T‐1, T‐2, T‐3, and T‐4 represent the data collected 1, 2, 3, and 4 years prior to the last assessment. Patients were followed for as long as they failed to progress to dementia, up to the end of the cohort study, with a maximum follow‐up of 94 months (average = 33.88 months).

### 2.2 Participants^1^

One‐hundred and fifty‐one participants were recruited from memory clinics and met the criteria^3^, ^29^ for amnestic MCI at entry. Thirty participants only had one assessment and were excluded from the analyses. Following study entry, participants received a yearly clinical follow‐up that allowed to identify those who had progressed, hereby progressors. Progressors (*N* = 47) were found to meet the clinical *DSM*‐*IV* criteria for dementia of the Alzheimer type^30^ at any point over the course of the follow‐up. Amongst the nonprogressors, some showed a significant cognitive decline (more than 1.5 *SD* from 1 year to the other) on neuropsychological tests and were thus classified as decliners (*N* = 16). It is hypothesized that these individuals are in an earlier stage of the disease process and had not yet reached the point at which they could meet the criteria for dementia over the course of the follow‐up. They were thus examined as a group of interest. The remaining of the nonprogressors were classified as stable MCI (*N* = 58).

### 2.3 Cognitive measures

Six neuropsychological tests were used to assess the cognitive profile of the participants: the RL/RI^31^ (Free‐Recall and Cued word Recall), the Rey complex geometrical figure test, 3 min‐delay score^32^ the Stoop‐Victoria test,^33^ the Coding‐subtest of the WAIS‐R^34^ the Benton Judgment of line orientation^35^ and the 15‐item version of the Boston Naming test.^36^ For a detailed description of the cognitive tests, see Cloutier et al.^18^ The inclusion of these neuropsychological tests in the cognitive battery was based on three criteria: (1) they are standard tasks used in clinical setting; (2) they were shown to be sensitive in detecting cognitive impairment associated with AD^37^; and (3) they cover multiple cognitive domains, mainly episodic memory, executive functions, working memory, language, and visuospatial processing.

### 2.4 Instrumental activities of daily living

The instrumental subscale of the *Système de mesure de l'automie fonctionnelle* (SMAf; a French‐language functional autonomy questionnaire) was used to assess performance in iADL. This self‐reported scale was chosen since it was shown to have good inter‐rater agreement and test‐retest reliability.^38^ It includes eight iADL areas (cleaning, cooking, shopping, laundry, telephone use, use of transportation, in medication intake, and budget management) Each area is comprised of one item/question, scored by the participant between 0 and 3 (0 representing no self‐reported impairment and 3 a significant handicap; e.g., for the budget item: 0 = Can manage budget alone; 1 = Needs help with major transactions; 2 = Needs help with daily transactions but is able to use pocket money; and 3 = Cannot manage a budget). Thus, scores on individual item range from 0 to 3 and total scores range from 0 to 24. The clinical classification and dementia diagnosis of the participants were independent from the results of the SMAf instrument.

### 2.5 Analysis

We determined the outcomes by assessing which SMAf‐8 items were grouped into subdomains of iADL with a principal components analysis on the scores obtained for each item on T0, using data from the whole group. This was done because it was expected that different iADL domains would have a different progression trajectory.

We then ran polynomial regression analyses (growth curves, mixed linear model analysis) to determine which model best fits the data over the 5‐year follow‐up period. This was done for each group (progressors, decliners, and stable) separately. The dependent variables were the total score on the SMAf (0–24) and the average score for the items clustering on the PCA determined factors. The data was analyzed as a function of time to diagnosis. We first verified whether a linear model was significant for each group and if it was, we proceeded to test more sophisticated models: a quadratic function, a second‐order polynomial characterized by one fracture in the curve and the cubic function, a third‐order polynomial. We also included age, gender, and education as controlled variables in the model. Time was considered as repeated effects with a compound symmetry correlation matrix.

Following the regression analyses, we used a separate 2 (Groups: progressors, nonprogressors) × 3 (Time: T0, T‐1, T‐2) mixed analyses of variance (ANOVA) for each dependent variable to identify more precisely at what time the groups differed from one another. Here, the stable MCI group and the decliners were combined into a nonprogressor group, so that the sample meets the postulate for group comparisons using parametric analysis. Only 3 years were used in order to maximize the number of participants, as ANOVA is not resistant to missing data, and it was done with only the participants with at least 3 years of follow‐up prior to diagnosis. The adjusted *F* was used to correct sphericity by removing the part of the effect that is explained by the systematic error. Greenhouse–Geisser's estimates were used to correct for error of the first kind.

Finally, we assessed the predictive accuracy of the models, meaning the predictive accuracy of future progression to dementia, by combining logistic regressions (Wald backward elimination stepwise selection) with receive operating characteristic (ROC) curve analyses. The cognitive measures as well as the iADL scores were entered as predictors. The regressions were performed on each of the 3 years prior to dementia conversion.

## 3 RESULTS

### 3.1 Socio‐demographic and clinical characteristics

The data from 121 participants (74 women) was analyzed. Demographic and clinical data are presented in Table 1.

**TABLE 1:** Clinical and demographic characteristics at entry and on T0 (mean, *SD* in parentheses)

	Stable	Decliners	Progressors	*p*
Age				
At entry	68.09 (9.2)	74.63 (6.4)	71.47 (7.3)	**0.02**
On T0^a^	72.53 (9)	76.43 (6.4)	74.11 (7.3)	0.21
Education (years)	14.69 (3.9)	14.31 (5.3)	14.23 (4.1)	0.85
*N*(men/women)	58 (22/36)	16 (8/8)	47 (17/30)	0.61
Length of follow‐up (months)	39.98 (17.7)	20.88 (12.6)	30.77 (19.6)	**<0.01**
GDS				
At entry	1.23 (1.2)	1.5 (1.3)	1.05 (1.3)	0.46
On T0	1.26 (1.3)	1.4 (1.5)	1.21 (1.1)	0.89
MMSE on T0	28.08 (2)	27.47 (1.9)	26.07 (2.6)	**<0.01**
MATTIS on T0	137.52 (2.9)	130.47 (8.2)	126.17 (10.1)	**<0.01**

Abbreviations: GDS, geriatric depression scale; MATTIS, mattis dementia rating scale; MMSE, mini mental state examination; PCA, principal component analysis; WAIS‐R, wechsler adult intelligence scale‐revised.

^a^ Corresponds to the year of dementia progression in progressors and last year of testing for decliners and stable.

Values in bold are statistically significant (*p* < 0.05).

### 3.2 Principal component analysis for iADL

When 2 components are included, the proportion of variance explained is 57.64%. The items that cluster on Component 1 are telephone use, medication intake, use of transportation, budget management, and shopping, suggesting that Component 1 reflects the ability to carry on “complex iADL.” The items cleaning, laundry, and cooking loaded on Component 2, which was interpreted as reflecting a general “housekeeping iADL” factor.

### 3.3 Growth curve models for total iADL

**FIGURE 1:**
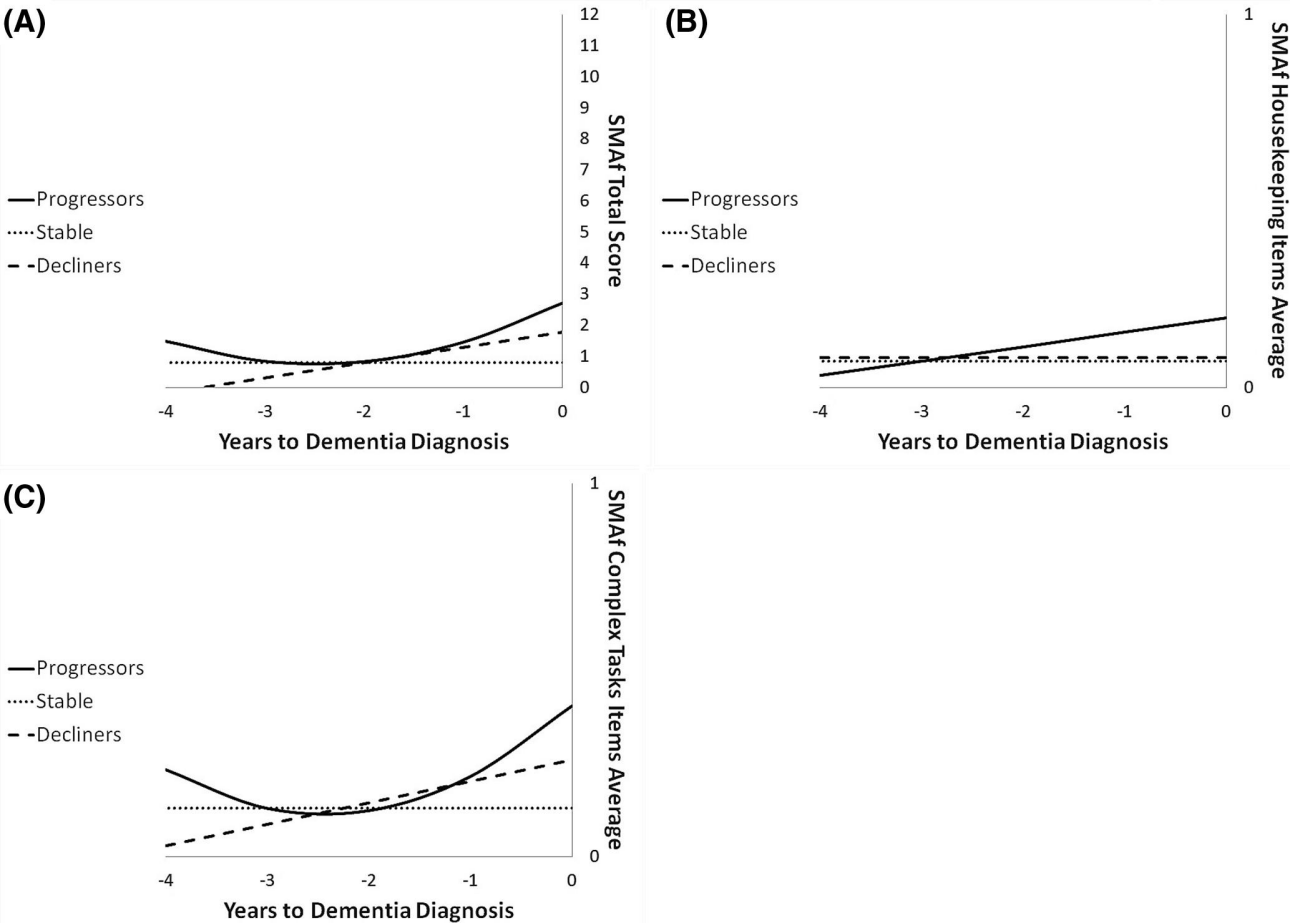
Trajectories of decline in stable (dotted lines), decliners (broken line), and progressors (full line) as a function of time to diagnosis on (A) total iADL, (B) housekeeping, and (C) complex iADL. (A) Score on SMAf total (sum of the eight items) as a function of time to diagnosis (for progressors) or on the last five cognitive assessments (for the decliners and stable). Note that a higher score represents more functional impact. A quadratic function best describes the distribution for the progressors: black line. A linear function best describes the distribution for the decliners: big dots line. No significant model for stable: small dots line. (B) Score on the housekeeping‐related iADL items as a function of time to diagnosis (for progressors) or on the last five cognitive assessments (for the decliners and stable). Note that a higher score represents more functional impact. A linear function best describes the distribution for the progressors: black line. No significant model for the declines (big dots line) and the stable: small dots line. (C) Score on the complex iADL items as a function of time to diagnosis (for progressors) or on the last five cognitive assessments (for the decliners and stable). Note that a higher score represents more functional impact. A quadratic function best describes the distribution for the progressors: black line. A linear function best describes the distribution for the decliners: big dots line. No significant model for stable: small dots line. iADL, instrumental activities of daily living

The regression analysis for total iADL indicated a significant quadratic trend for the progressors and a significant linear trend for the decliners (see Figure 1A). None of the models were found significant for the stable group. The ANOVA on the total iADL score indicated a significant Group × Time interaction (*F* (2, 122) = 14.83, *p* < 0.05; partial eta squared = 0.2). The interaction was due to Time being significant for progressors (*N* = 24; *F* (2, 46) = 10.43, *p* < 0.05; partial eta squared = 0.31), but not for the nonprogressors (*N* = 39). Post‐hoc comparisons with Bonferroni adjustments in progressors indicate that T0 (*M* = 3.21) differed from T‐1 (*M* = 1.67) and T‐2 (*M* = 1.04), but T‐1 and T‐2 did not differ from each other. Furthermore, the progressors had a significantly higher score for total iADL impairment than nonprogressors on both T0 (mean difference = 2.27; partial eta squared = 0.27) and T‐1 (mean difference = 0.82; partial eta squared = 0.17).

### 3.4 Growth curve models for housekeeping‐related iADL

The regression analysis for *Housekeeping‐related iADL* indicated a significant linear trend for the progressors (see Figure 1B). None of the models were found significant for the stable group. The ANOVA on *housekeeping‐related iADL* indicated a significant Group × Time interaction (*F* (2, 106) = 6.3, *p* < 0.05; partial eta squared = 0.11). The interaction was due to Time being significant for progressors (*N* = 21; *F* (2, 40) = 4.06, *p* < 0.05; partial eta squared = 0.17), but not for nonprogressors (*N* = 34). Furthermore, post‐hoc comparisons indicated that the progressors had a significantly higher score for housekeeping‐related iADL than nonprogressors on T0 only (mean difference = 0.22; partial eta squared = 0.14).

### 3.5 Complex iADL

The regression analysis for *complex iADL* indicated a significant quadratic trend (relative stability followed by a rapid decline) for the progressors and a significant linear trend for the decliners (see Figure 1C; note that a higher score represents more impairment on daily functioning). None of the models were found significant for the stable group. The ANOVA on *complex iADL* indicated a significant Group × Time interaction (*F* (2, 106) = 6.51, *p* < 0.05; partial eta squared = 0.11). Time was significant for the progressors (*N* = 21; *F* (2, 40) = 5.27, *p* < 0.05; partial eta squared = 0.21), but not for nonprogressors (*N* = 34). Post‐hoc comparisons with Bonferroni adjustments in progressors indicated that T0 (*M* = 0.43) differed from T‐2 (*M* = 0.17). Furthermore, the progressors had a significantly higher score for complex iADL impairment than the nonprogressors on both T0 (mean difference = 0.27; partial eta squared = 0.17) and T‐1 (mean difference = 0.11; partial eta squared = 0.16).

**TABLE 2:** Logistic regressions for dementia conversion prediction

	B (SE)	*p*
T‐3		
Constant	2.697 (0.615)	**0.000**
Delayed word recall	−0.384 (0.072)	**0.000**
T‐2		
Constant	0.613 (1.249)	0.624
Delayed word recall	−0.353 (0.113)	**0.002**
Stroop inhibition	0.143 (0.053)	**0.007**
T‐1		
Constant	3.054 (1.337)	**0.022**
Delayed word recall	−0.366 (0.085)	**0.000**
Stroop inhibition	0.06 (0.026)	**0.019**
Coding	−0.249 (0.119)	**0.037**
Complex iADL	4.477 (2.169)	**0.039**

Values in bold are statistically significant (*p* < 0.05).

### 3.6 Predictive diagnostic accuracy

**FIGURE 2:**
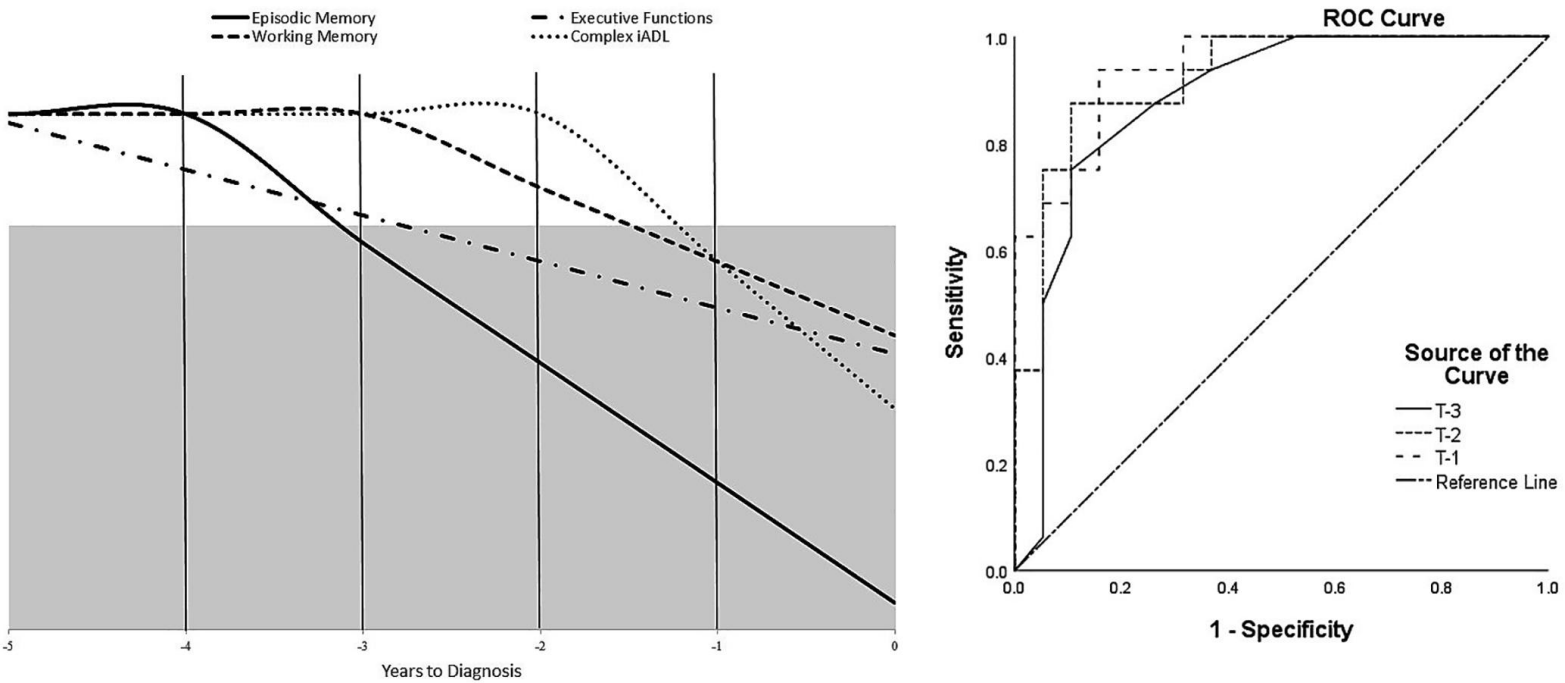
Prediction model of progression from MCI to dementia (left) and diagnostic accuracy of this model (right). The grey area represents the significant predictors of conversion, based on the logistic regression analysis. ROC curve analysis indicated a good accuracy 3 years prior the diagnosis (AUC = 0.88 [95% confidence level; lower bound: 0.76, upper bound: 1]) and an excellent accuracy 2 years (AUC = 0.92 [95% confidence level; lower bound: 0.84, upper bound: 1]) and 1 year (AUC = 0.94 [95% confidence level; lower bound: 0.87, upper bound: 1]) before diagnosis. AUC, area under the curve; MCI, mild cognitive impairment; ROC, receive operating characteristic

The results of the logistic regression analysis (see Table 2) indicated that 3 years prior to dementia conversion (T‐3), only the score of the delayed word recall was a significant predictor of dementia progression. On T‐2, both delayed word recall and cognitive inhibition (Stroop) were significant predictors, whereas on T‐1, the significant predictors of dementia progression were delayed word recall, cognitive inhibition, working memory (Coding), and *complex iADL*. By combining the results of the logistic regression analysis with the trajectories of decline found using the polynomial regression analysis, we proposed a theoretical model of progression from MCI to dementia (see Figure 2).

The predictive accuracy of this model was assessed using ROC curve analysis (see Figure 2). Three years prior to the diagnosis, the performance of delayed word recall alone has a good predictive accuracy (area under the curve [AUC] = 0.88). The predictive accuracy increases to excellent (AUC = 0.92) 2 years prior to dementia progression when we include the performance on the executive functions task (cognitive inhibition). The diagnosis accuracy is also very high (AUC = 0.94) the year preceding dementia conversion when we include the significant predictors (episodic memory, executive functions, working memory, and complex iADL scores).

## 4 DISCUSSION

The goal of this study was to determine the decline trajectories of iADL in MCI in the years preceding a diagnosis of dementia. The study differs from most longitudinal studies reporting iADL impairment in MCI, by analyzing the data as a function of time to diagnosis rather than study entry, and by examining complex models of decline in addition to the more traditional linear model.

A significant proportion of the participants (39%) progressed to dementia during the course of the study. MCI participants who progressed to dementia started to experience a significant decline in their total iADL score about 2 years prior to the year they received their diagnosis. As a result, the impairment on iADL was apparent and statistically significant at least 1 year prior to their diagnosis. Even though the groups did not differ 2 years prior the progressors's diagnosis, a decline was nevertheless observed during that time. Indeed, we found that the trajectory followed a quadratic trend. In other words, scores on iADL remained stable for many years before presenting the rapid decline found in the 2 years before diagnosis.

A group of participants were identified as decliners. Those MCI did not meet the clinical criteria of dementia during the follow‐up of the study, but still showed a significant cognitive decline over the years. They nevertheless experienced a decline in iADL but it was slower and more gradual than in those who received their diagnosis within the timeframe of this study. It is likely that decliners are in an earlier phase of the disease than progressors. Thus, the linear decline might represent a prior state which we may not have been able to observe in progressors as their assessment may not have extended far back enough.

Importantly, the two iADL categories progress differently during the MCI phase. The trajectory of decline found for the total iADL score and described above seems to be explained by complex iADL. Indeed, this category of iADL follows the exact same trajectory, that is a quadratic trend for progressors, a linear trend for decliners and no effect of time for the stable MCI.

The results of the component principal analysis identified two broad iADL categories: housekeeping‐related activities (cleaning, cooking, and laundry) and complex iADL (telephone use, medication intake, use of transportation, budget management, and shopping). This complex iADL category contains activities that have been related to cognitive decline and were found to be predictive of conversion to dementia the following year in a large longitudinal population study.^39^, ^40^ This category of iADL is also very similar to the Barberger‐Gateau's 4‐iADL (telephone use, transportation, medication intake, and budget management). A functional impairment on these 4‐iADL was suggested, in a population cohort, to represent an early marker of incident dementia up to 3 years before the diagnosis.^41^ Our finding that the same domains are impaired with a very similar timeline in a clinical cohort is important because it indicates that the effect is independent of recruitment source and characteristics. Thus, the component analysis used here appears to have identified a clinically and empirically valid distinction among iADL, which brings external support to our approach.

Compared to other longitudinal studies^24^, ^25^, ^26^ reporting the functional decline in the preclinical phases of AD, our study specifically assessed the trajectories of iADL impairment in a clinical cohort of MCI individuals, by comparing those who progressed to AD with those who did not progress and remained stable. This approach allowed us to identify, among at‐risk individuals, the predictors associated with a true neurodegenerative process, beyond objective cognitive impairment.

One of our goals was to combine the complex iADL scores with the performance in cognition^20^ to assess the diagnostic accuracy of a prediction model of progression from MCI to dementia. We observed that a significant impairment of episodic memory predicts progression 3 years prior to the diagnosis. We obtain an excellent diagnostic accuracy 2 years before dementia progression by combining the performance on both memory and executive functions task (significant predictors). This is unsurprising since we know that episodic memory and executive functions are both impaired early in the disease process and are predictors of conversion from MCI to AD.^28^, ^42^ Cognitive tests have been shown to be excellent at predicting which MCI individuals will progress to dementia, and the predictive accuracy seems to be the highest when combining memory measures with a small set of other domains.^43^ Our model reflects these findings since multi‐domain impairment increases the risk of MCI to dementia conversion.

The year prior to the diagnosis, we observe that, on top of memory and executive functioning impairment, a decline in working memory and complex iADL contributes to predict progression. Thus, changes on complex iADL are accompanied by changes in working memory and preceded by a significant decline in executive functioning. This is consistent with data showing that executive functions are good predictors of functional impact in patients with relatively mild dementia,^44^ in patients with frontal lesions and in community‐dwelling older adults.^45^, ^46^ Our findings on the relationship between cognitive impairment and the decline in iADL, particularly for complex tasks, support the already established evidence from the literature. Our model proposes that a change in the ability to perform complex iADL may signal imminent conversion the following year. This is clinically relevant and may help the clinicians to implement interventions and accommodations as early as possible for their at‐risk patients and their family.

The strength of this model is that it relies on a very simple clinical assessment which is relatively cheap and readily available for family doctors' practice. This is a notable advantage over more complex investigations that include imaging and biomarkers.

### 4.1 Limitations

Some limitations must be acknowledged. First, the diagnosis was based on clinical criteria and we did not include biomarkers. For this reason, we are unable to draw conclusions regarding the etiology of the disease in these individuals.^1^, ^47^ Second, we did not include healthy older adults to serve as a control group, as our goal was to examine the natural history of a clinical cohort as a function of future progression to dementia. As a result, it is not possible to know whether nonprogressors (stable/decliners) MCIs are impaired relative to a comparative group of older adults with no complaint. It is of note, however, that when comparing mean performance levels to those provided by normative values, stable MCI participants present a performance very similar to that of healthy older adults, apart from verbal memory which is impaired by design.

To measure everyday functioning, we used an 8‐item self‐reported questionnaire. Though this has the advantage of simplicity of use, it may be biased by the participant's own impression of their abilities. Furthermore, the ecological validity of the measure is not assured as it is not a performance‐based score, but rather a self‐reported one. It is possible that the degree of daily functioning interference expressed by the MCI progressors may not be entirely accurate and may be underestimated, since anosognosia was reported in MCI.^48^ However, other studies^49^ seem to suggest that most MCI patients tend to overestimate deficits when compared to a caregiver's assessment, while AD patients in early stages of the disease may underestimate their deficits. In this study, cognitively stable MCI nonprogressors did not report any changes for their ability to perform iADL, whereas the MCI participant who effectively received a probable AD diagnosis did report a decline in iADL. Thus, this decline seems to represent a valid marker of true AD progression and may help to identify the MCI individuals who are more at risk of dementia conversion. Also, the questionnaire may lack sensitivity to subtle difficulties expressed by MCI participants. Note however that we did nevertheless observe a reasonable range of values and were able to derive statistically valid models. Furthermore, the SMAf instrument was shown to have an excellent test‐retest reliability and a good inter‐rater reliability, which was further improved with revisions made to the scale.^38^, ^50^ It also has an excellent criterion validity, the score on the SMAf being highly correlated with the number of hours of care provided to the patient.^50^

It is important to mention that the physicians certainly questioned the patients regarding changes on their ability to perform complex iADL in their assessment, which may indicate an intercorrelation between the clinical interview and the SMAf scores. However, the SMAf instrument was not used explicitly and systematically for the dementia diagnosis. Thus, the results still provide an external support for the clinical validity of assessing complex iADL.

Finally, even though the decline in iADL functioning was characterized by stability followed by a rapid decline just prior to AD diagnosis in the MCI progressors, it could be the case that iADL functioning declines many years before the onset of dementia, perhaps very gradually, but that this was not captured by the SMAf, especially since the complex iADL is comprised of only five items. This gradual and subtle decline was perhaps captured in the decliners group, which we hypothesized to be in an earlier phase of the disease.

### 4.2 Conclusion and implications

This study provides new information regarding the trajectory of iADL change during the MCI phase. Most prior studies have assumed a linear change function and change scores are typically derived using formula that don't consider the trajectory of change. Here, we found that complex iADL are characterized by a quadratic function, that is years of stable performance followed by a decline just prior to dementia progression. This highlights the importance of including iADL in the evaluation of MCI individuals and further challenges the idea that performance on activities of daily living is intact and does not change during the MCI phase.

## CONFLICT OF INTEREST

The authors have no relevant conflict of interest to disclose.

## Data Availability

The data that support the findings of this study are available from the corresponding author upon reasonable request.

## ENDNOTE

^1^ The same cohort of participants was part of a previous study investigating the patterns of cognitive decline prior to dementia.^18^
